# Genetic and functional study of StAR (steroidogenic acute regulatory protein) deficiency

**DOI:** 10.1186/1687-9856-2015-S1-O8

**Published:** 2015-04-28

**Authors:** Chan Jong Kim

**Affiliations:** 1 Department of Pediatrics, Chonnam National University, Medical School & Hospital, Gwangju, South Korea

## 

Congenital lipoid adrenal hyperplasia (lipoid CAH) is the most severe form of CAH, impairing adrenal and gonadal steroidogenesis. Most cases of lipoid CAH are caused by recessive mutations in the gene encoding steroidogenic acute regulatory protein (StAR), a protein that plays an essential role in cholesterol transfer from the outer to inner mitochondrial membrane, thus providing the substrate for steroid hormone biosynthesis. Affected children typically present with life-threatening adrenal insufficiency in early infancy due to a failure of glucocorticoid (cortisol) and mineralocorticoid (aldosterone) biosynthesis, and 46,XY genetic males have complete lack of androgenization and appear phenotypically female due to impaired testicular androgen secretion in utero. Previously, the Q258X mutation of StAR was shown to account for about 70% of affected alleles in most patients of Japanese and Korean ancestry. However, it is more prevalent (92.3%) in the Korean population. These results suggest that the genetic defect in the StAR gene in Korean patients with lipoid CAH is highly homogeneous, probably reflecting a founder effect. Recently, some patients have been reported that they showed late and mild clinical presentation. These cases and studies establish a new entity of ‘non-classic lipoid CAH’ showing that the phenotypic spectrum of StAR mutations is substantially broader than previously appreciated.

